# Spatial and Temporal Virus Load Dynamics of SARS-CoV-2: A Single-Center Cohort Study

**DOI:** 10.3390/diagnostics11030427

**Published:** 2021-03-03

**Authors:** Enagnon Kazali Alidjinou, Julien Poissy, Mahdi Ouafi, Morgan Caplan, Ilyes Benhalima, Julien Goutay, Claire Tinez, Karine Faure, Marie-Charlotte Chopin, Cécile Yelnik, Marc Lambert, Didier Hober, Sébastien Preau, Saad Nseir, Ilka Engelmann, on behalf of the Lille COVID Research Network (LICORNE)

**Affiliations:** 1 Laboratoire de Virologie, Univ Lille, CHU Lille, ULR3610, F-59000 Lille, France; EnagnonKazali.ALIDJINOU@CHRU-LILLE.FR (E.K.A.); Mahdi.OUAFI@CHRU-LILLE.FR (M.O.); Ilyes.BENHALIMA@CHU-LILLE.FR (I.B.); Claire.TINEZ@CHRU-LILLE.FR (C.T.); Didier.HOBER@CHRU-LILLE.FR (D.H.); 2 Pôle de réanimation, CNRS, UMR 8576-UGSF-Unité de Glycobiologie Structurale et Fonctionnelle, Univ. Lille, CHU Lille, Inserm U1285, F-59000 Lille, France; Julien.POISSY@chu-lille.fr (J.P.); Morgan.CAPLAN@CHRU-LILLE.FR (M.C.); Julien.GOUTAY@CHRU-LILLE.FR (J.G.); Sebastien.PREAU@CHRU-LILLE.FR (S.P.); Saadalla.NSEIR@CHRU-LILLE.FR (S.N.); 3 Service de Maladies infectieuses, CHU Lille, F-59000 Lille, France; Karine.FAURE@CHRU-LILLE.FR (K.F.); Mariecharlotte.CHOPIN@CHRU-LILLE.FR (M.-C.C.); 4 Service de Médecine Polyvalente de Post-Urgence, Univ. Lille, CHU Lille, F-59000 Lille, France; Cecile.YELNIK@CHRU-LILLE.FR (C.Y.); Marc.LAMBERT@CHRU-LILLE.FR (M.L.)

**Keywords:** COVID-19, SARS-CoV-2, viral shedding, molecular diagnostics, RT-PCR

## Abstract

The severe acute respiratory syndrome coronavirus 2 (SARS-CoV-2) has caused an ongoing pandemic. Reverse transcription polymerase chain reaction (RT-PCR) is the gold standard for the detection of SARS-CoV-2 and has been applied to different specimen types. Understanding the virus load and virus detection frequency in different specimen types is important to improve diagnosis and estimate the duration of potential infectivity. We conducted a retrospective single-center cohort study on hospitalized and outpatients with SARS-CoV-2 infection. We analyzed the frequency of virus detection, virus load, and duration of the virus excretion in upper and lower respiratory specimens as well as stool and plasma. We found that the frequency of SARS-CoV-2 detection, the virus load, and duration of virus excretion was higher in lower respiratory tract (LRT) than in upper respiratory tract (URT) specimens. The duration of virus excretion was longer in patients requiring intensive care unit (ICU) admission. In conclusion, LRT specimens are the most appropriate specimen type for the detection and follow-up of SARS-CoV-2 infection. Duration of virus excretion is longer in severe cases of SARS-CoV-2 infection.

## 1. Introduction

A novel coronavirus, later named SARS-CoV-2, associated with severe acute respiratory syndromes, emerged in Wuhan, China at the end of 2019 [1,2] and rapidly spread worldwide to cause a pandemic with over 77 million cases and 1.7 million deaths reported as of 22 December 2020 [3]. Virological diagnosis is based on detection of viral RNA by RT-PCR [4]. Several laboratories have developed RT-PCR assays available to perform SARS-CoV-2 RNA detection. The optimal specimen type for diagnosis has yet to be determined. Upper respiratory tract (URT), lower respiratory tract (LRT), and stool specimens have been suggested to be suitable for this purpose [4,5]. However, a detailed knowledge of the temporo-spatial virus load kinetics is necessary to determine which specimen type is the most useful for diagnosis. Since the beginning of the pandemic, a lot of studies have been published on small case series or small patient cohorts focusing mainly on the kinetics of viral detection in hospitalized patients with SARS-CoV-2 [6,7,8,9], reviewed in [10].

Furthermore, the duration of infectivity of SARS-CoV-2-infected patients is highly relevant to adapt infection control measures. Therefore, a detailed analysis of the duration of virus excretion is of importance. In our study, we reported data of a large cohort of patients with laboratory-confirmed SARS-CoV-2 infection. We analyzed 2008 specimens of 520 patients with SARS-CoV-2 infection who were either admitted to Lille University hospital or treated as outpatients. We presented a detailed analysis of the frequency of SARS-CoV-2 RNA detection, virus load profiles in different specimen types including upper and lower respiratory tract specimens, stool, and plasma, and duration of virus excretion depending on the time after symptom onset.

## 2. Materials and Methods

### 2.1. Study Design

A retrospective cohort study of patients with laboratory-confirmed SARS-CoV-2 infection from 26 February 2020 to 2 May 2020 was conducted at the Lille University Hospital Center (CHU Lille). Demographic data and data of specimens sent for routine diagnostic purposes were retrospectively collected from hospital charts and the virology laboratory database.

This study was approved by the French Institutional Authority for Personal Data Protection (Commission Nationale de l’Informatique et des Libertés DR-2020-178, 22 October 2020) and the ethics committee (ECH20/09, 7 September 2020).

### 2.2. SARS-CoV-2 RT-PCR

Due to the high specimen number and shortages in the supply of kits and consumables for extraction and RT-PCR, different commercially available kits and a laboratory-developed RT-PCR were used for the initial diagnosis of SARS-CoV-2 infection. These were AllPlex 2019-nCoV Assay (Seegene, Seoul, Korea), VIASURE SARS-CoV-2S gene Real-time PCR Detection Kit (CerTest, Zaragoza, Spain), and Realstar SARS-CoV-2 RT-PCR Kit1.0 (Altona Diagnostics, Hamburg, Germany). Commercially available kits were used in accordance with the manufacturers’ instructions. Specimens were handled in a biosafety level 2 facility in a class II biological safety cabinet as recommended by the World health organization [11]. Additionally, personnel protective equipment was used including disposable laboratory clothing, wrap-around gowns, FFP2 masks, hair caps, two pairs of disposable gloves, and eye protection. RNA extraction was either performed in the biosafety level 2 facility for specimens analyzed with the laboratory developed assay (see below) and the VIASURE SARS-CoV-2S gene Real-time PCR Detection Kit. For specimens analyzed with assays that included automatic RNA extraction (AllPlex 2019-nCoV Assay and Realstar SARS-CoV-2 RT-PCR Kit 1.0), lysis buffer was added to the specimens in the biosafety level 2 facility and specimens were further processed outside the biosafety level 2 facility.

In addition, a laboratory-developed real-time RT-PCR method developed by the French Reference Center for respiratory viruses (Institut Pasteur, Paris) was used for SARS-CoV-2 RNA detection [12]. It is a duplex RT-PCR targeting two regions in the *RdRp* gene, named IP2 and IP4. *G6PDH* RT-PCR using primers G6PDH-6 (GAAGGTGAAGGTCGGAGT) and G6PDH-231 (GAAGATGGTGATGGGATTTC), and the probe G6PDH-202 (5′FAM-CAAGCTTCCCGTTCTCAGCC-3′BHQ) was additionally performed to monitor for specimen quality, RNA extraction, and PCR inhibition. Specimens with negative SARS-CoV-2 RT-PCR and undetectable *G6PDH* were reanalyzed after a new round of RNA extraction. If *G6PDH* was again undetectable, the result was considered uninterpretable. This was the case in 4% of specimens and 62% of these were stool specimens. Specimens with uninterpretable results were excluded from the analysis.

Follow-up specimens were analyzed using this laboratory-developed real-time RT-PCR assay exclusively. Ct values were used to analyze virus loads. Ct values were inversely correlated to the quantity of RNA target present in the specimen.

### 2.3. Definitions

URT specimens were nasopharyngeal swabs. Flocked swabs were used to obtain nasopharyngeal swab specimens and placed in universal transport medium (UTM). LRT specimens included tracheal or tracheo-bronchial aspirations and bronchoalveolar lavage specimens.

The minimum duration of virus detection was defined as the time period from symptom onset to the last positive specimen of the same specimen type. Maximum duration of virus detection was defined as the time period from symptom onset to the first negative specimen (obtained after the last positive specimen) of the same specimen type.

### 2.4. Statistical Analysis

IBM SPSS Statistics for Windows, Version 22.0 (IBM Corp, Armonk, NY, USA) and GraphPad Prism (GraphPad Software, San Diego, CA, USA) were used for statistical analyses. Fisher’s exact test was used to compare categorical variables. Non-parametric tests were performed to compare quantitative variables (Mann–Whitney U test or Kruskal–Wallis test as appropriate). The correlation of virus load with the time after symptom onset was analyzed by Spearman correlation. A *p*-value of <0.05 was considered statistically significant.

## 3. Results

Five hundred and twenty patients with laboratory-confirmed SARS-CoV-2 infection were included in the study. Fifty-seven percent were male, and the median age was 57 years (range 0 to 94 years). Three hundred and eighty-six patients (74%) were admitted to the hospital and 191 (37%) to the ICU. Two hundred and eighty-nine (55%) required oxygen support. A total of 2008 specimens of these patients were tested for SARS-CoV-2 RNA by RT-PCR: 990 URT specimens, 687 LRT specimens, 263 stool specimens, and 68 plasma specimens. Four hundred and thirty-three patients had at least one URT specimen (median 2; range 1–9), 333 patients had at least one LRT specimen (median 1; range 1–8), 133 had at least one stool specimen (median 2; range 1–7), and 54 patients had at least one plasma specimens (median 1; range 1–4).

### 3.1. Frequency of SARS-CoV-2 Detection

**Figure 1:**
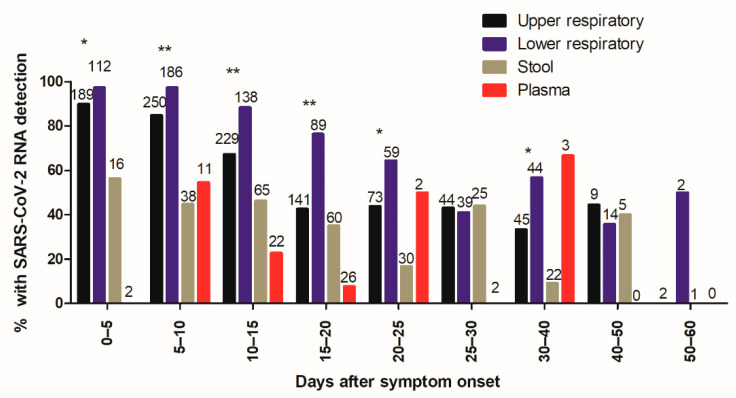
Frequency of SARS-CoV-2 RNA detection as a function of time after symptom onset.

Our results found that 383/433 (88.5%) of patients had SARS-CoV-2 RNA detected in the URT, 329/333 (98.8%) had SARS-CoV-2 RNA detected in the LRT, 67/133 (50.4%) had SARS-CoV-2 RNA detected in stool, and 14/54 (25.9%) had SARS-CoV-2 RNA detected in plasma. The percentage of specimens with detection of SARS-CoV-2 RNA in different specimen types depending on the time after symptom onset is shown in Figure 1. Frequency of SARS-CoV-2 RNA detection in the URT and LRT specimens gradually decreased from 90% and 97%, respectively, in the first 5 days after symptom onset to 43% and 76%, respectively, at 15 to 20 days after symptom onset. Interestingly, the decline in the percentage of positive URT specimens occurred earlier and was more pronounced than the decline in the percentage of positive LRT specimens.

The percentage of positive specimens was higher in LRT than in URT specimens for most time intervals (Figure 1). Frequency of SARS-CoV-2 RNA detection in stool and plasma specimens did not appear to be dependent on time after symptom onset, but this was not analyzed statistically because of the low number of specimens.

Figure 1 shows the percentage of specimens with detection of SARS-CoV-2 RNA in upper respiratory tract (URT) specimens (black bars), lower respiratory tract (LRT) specimens (blue bars), stool specimens (brown bars), and plasma specimens (red bars) depending on the time after symptom onset. Total numbers of specimens are indicated at the top of the bars. Statistically significant differences between URT and LRT specimens are indicated (* *p* < 0.05, ** *p* < 0.001).

### 3.2. Virus Load

**Figure 2:**
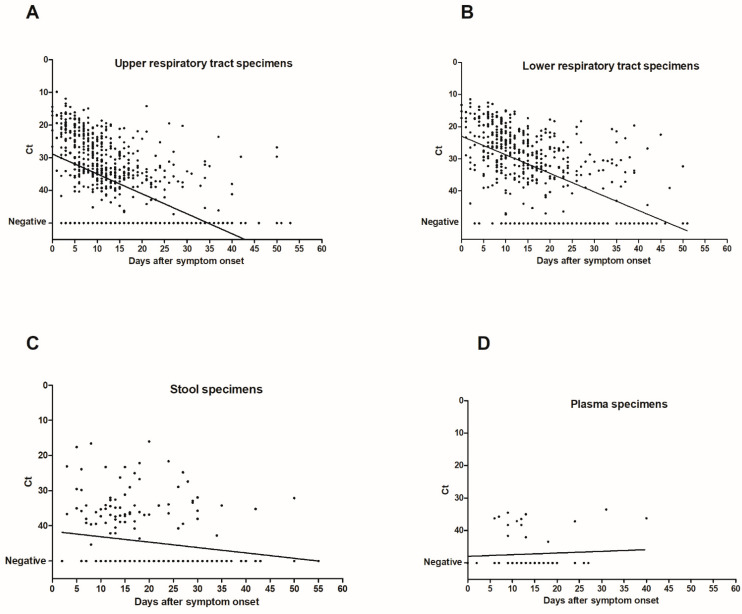
Correlation of Ct values with time after symptom onset.

**Figure 3:**
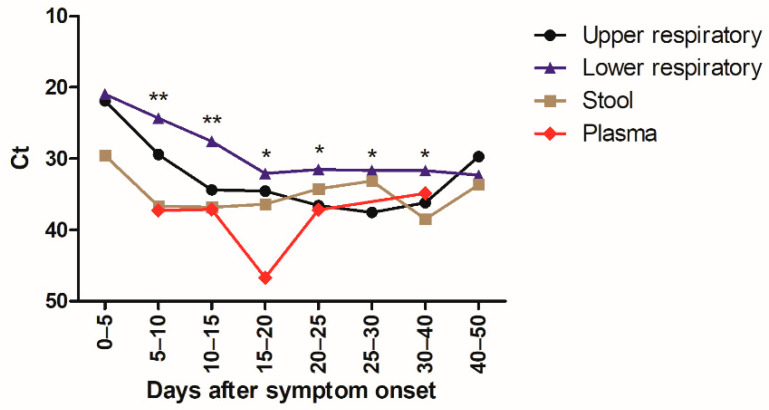
Kinetics of SARS-CoV-2 virus load as a function of time after symptom onset.

**Figure 4:**
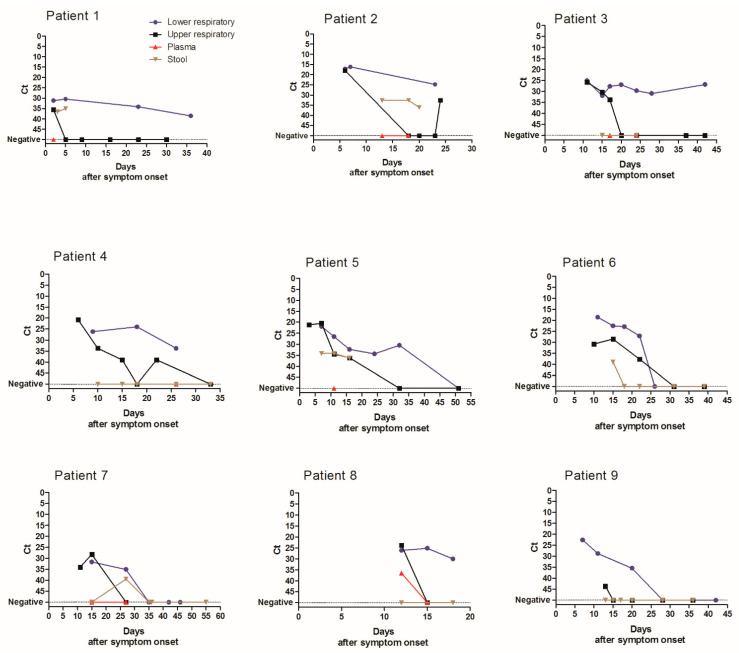
Spatio-temporal SARS-CoV-2 virus load kinetics in individual patients.

A total of 1547 specimens were analyzed with the same laboratory developed RT-PCR assay [12]. The virus load in URT, LRT, and stool specimens was inversely correlated with time between symptom onset and sampling (*p* < 0.0001 for URT and LRT specimens and *p* = 0.005 for stool specimens) (Figure 2). Median Ct values of positive specimens are shown in Figure 3. Ct values were lower corresponding to a higher virus load in LRT than in URT specimens for specimens taken between day 5 and 40 after symptom onset (Figure 3). In order to investigate whether the findings observed in the cohort are representative of the virus load kinetics in individual patients, nine patients who had several follow-up specimens of different specimen types were analyzed in detail. These patients were all admitted to ICU and needed mechanical ventilation. Patients’ characteristics can be found in Table S1. Virus load kinetics in URT, LRT, stool, and plasma specimens of these patients are shown in Figure 4. The finding that virus load is higher in LRT compared to URT specimens was confirmed for individual patients (Figure 4).

Figure 2 shows dot plots of Ct values (of target IP4) as a function of time after symptom onset in URT specimens (*n* = 724) (**A**), LRT specimens (*n* = 489) (**B**), stool specimens (*n* = 219) (**C**), and plasma specimens (*n* = 62) (**D**). There was an inverse correlation of Ct values with time after symptom onset (*p* < 0.0001 for URT and LRT specimens and *p* = 0.005 for stool specimens; Spearman *r* = 0.56, 95% confidence interval (CI95) 0.51–0.61 for URT specimens; Spearman *r* = 0.56, CI95 0.49–0.62 for LRT specimens; Spearman *r* = 0.19, CI95 0.05–0.32 for stool specimens). Ct values of specimens with undetectable SARS-CoV-2 RNA were set to 50. The linear regression lines are shown in the figure.

Figure 3 shows the median Ct values (for target IP4) of positive URT specimens (black) (*n* = 468), LRT specimens (blue) (*n* = 386), stool specimens (brown) (*n* = 80), and plasma specimens (red) (*n* =16) depending on the time after symptom onset. Statistically significant differences of virus load in URT versus LRT specimens were indicated (* *p* < 0.05; ** *p* < 0.001).

Figure 4 shows the median Ct value (for target IP4) of URT specimens (black), LRT specimens (blue), stool specimens (brown), and plasma specimens (red) depending on the time after symptom onset in individual patients. Ct values of specimens with undetectable SARS-CoV-2 RNA were set to 50.

### 3.3. Duration of Virus Excretion

Duration of virus excretion was longer for LRT compared to URT specimens in individual patients (Figure 4). Therefore, the analysis of virus excretion in different specimen types was extended to the whole cohort. The analysis of median duration of virus detection was restricted to patients who had a negative follow-up specimen after the last positive specimen. Because sampling was not performed daily, minimum (min) duration of virus detection was defined as the time period from symptom onset to the last positive specimen. Maximum (max) duration of virus detection was defined as period from symptom onset to the first negative specimen (Table 1). The median minimum and maximum durations of virus detection were longer in LRT (median min 16 and max 24 days after symptom onset) compared to URT specimens (median min 12 and max 17 days) (*p* < 0.001, Table 1). Duration of virus excretion in URT specimens was longer in patients admitted to ICU (median 13–19 days) compared to those not requiring ICU admission (median 8–13 days) (*p* < 0.001, Table 1). Findings were similar for the duration of virus excretion in LRT specimens (Table 1) but only reached statistically significance for the median minimum duration of virus excretion (Table 1).

Because it has been suggested that patients with low virus loads may no longer be contagious [13], the duration of virus detection with Ct values lower than 33 corresponding to high virus loads was investigated. In URT specimens, median minimum duration of virus excretion with Ct lower than 33 was 5 and 11 days in patients not admitted or admitted to the ICU, respectively (*p* < 0.001), and the median maximum duration of virus excretion with Ct lower than 33 was 12 and 16.5 days in patients not admitted or admitted to the ICU, respectively (*p* = 0.002, Table 1). For LRT specimens, median minimum duration of virus excretion with Ct lower than 33 was not significantly different between patients not admitted to ICU and the ICU ones (9.5 vs. 15 days, respectively) (*p* =0.10), but median maximum duration of virus excretion with Ct lower than 33 was shorter in non-ICU patients compared to ICU ones (11 vs. 20 days, respectively) (*p* = 0.006, Table 1).

## 4. Discussion

We reported the detailed virological analysis of a large cohort of SARS-CoV-2-infected patients with longitudinal follow-up in different specimen types. In our study, the percentage of positive specimens was higher in LRT than in URT specimens before 25 days after symptom onset (Figure 1). This implies that LRT specimens are more reliable for the diagnostic of SARS-CoV-2 infection as has been suggested previously [4], especially in patients with pneumonia that occurs some days after the onset of the infection when the frequency of virus detection in the URT has already decreased. SARS-CoV-2 RNA was detected in stool in 50% of patients which is in the same range as recent meta-analyses where the pooled positivity rate was 41–44% of patients [14,15]. The percentage of positive stool specimens and the virus load were relatively stable with increasing time after the symptom onset, but specimen numbers were relatively low for these specimen types. SARS-CoV-2 RNA was detected in the plasma of 26% of the patients that had a plasma specimen analyzed. This percentage is slightly lower than reported by two recent studies, which found RNAemia in 33 to 38% of patients [16,17]. Ct values of positive plasma specimens were all higher than 33, showing that the load in plasma specimens is low. A recent study reported similar findings and demonstrated that no viable virus could be isolated from serum specimens [18]. Taken together, these results suggest that the risk of transmission of SARS-CoV-2 to laboratory staff by handling blood specimens for routine diagnostic purposes is negligible when routine safety procedures are followed. Not only the percentage of positive specimens, but also the virus load was higher in LRT specimens than in URT specimens both in individual patients (Figure 4) and the whole cohort (Figure 2 and Figure 3), confirming the finding reported by Wölfel and colleagues [8]. We observed the highest virus loads in respiratory specimens in the first days after symptom onset (Figure 2A and Figure 3), which is consistent with the results of other studies [6,7,8,19] and a recent review [20]. The decline of the virus load depending on time after symptom onset has been analyzed in a recent meta-analysis. Our findings are similar, resembling the data of moderate–severe patients in the meta-analysis [10].

A few studies have investigated the duration of virus excretion; however, the definition of the duration of virus excretion was not always clearly described. We chose to define the minimum duration of virus detection as the time period from symptom onset to the last positive specimen and the maximum duration of virus detection as the time period from symptom onset to the first negative specimen. Therefore, only patients with a negative follow-up specimen after the last positive one of the same specimen type were included in this analysis. The duration of virus detection in our study was similar to the one described by Zheng and colleagues. However, in our cohort, duration of virus detection was longest in LRT specimens (Table 1) [21]. The median minimum and maximum durations of virus detection in LRT specimens were 16 and 24 days, which was consistent with the median duration reported by others [21,22,23]. Duration of SARS-CoV-2 RNA detection was longer in LRT specimens than in URT specimens in our study as reported by a recent review and meta-analysis [10,20]. Taken together, these findings suggest that LRT specimens are most reliable for the diagnosis and follow-up of SARS-CoV-2 infection.

Concerning the association of duration of virus excretion and disease severity, there are contradictory findings in different studies [20,23]. Of note, in our cohort, patients requiring ICU care had a longer duration of virus excretion, suggesting that patients with severe infection have a longer duration of virus excretion.

Recently, it was suggested that patients with low virus load in respiratory specimens may no longer be contagious [12,23]. This prompted us to analyze the duration of virus excretion with Ct values lower than 33, corresponding to relatively high virus loads. The duration of virus excretion with Ct values lower than 33 was also longer in ICU patients. This suggests that there is a prolonged excretion of high virus loads and thus potentially a longer duration of infectivity in critically ill patients. This finding is of high relevance for the implementation and discontinuation of isolation measures of ICU patients. Whereas most specimens taken more than 21 days after symptom onset were negative in the study by He and colleagues [6], we still found virus detection for more than 40 days, especially in respiratory specimens. Although RT-PCR cannot distinguish between viable virus and viral RNA, the finding that some Ct values were low even 30 days after symptom onset is suggestive of persistent viral replication. Two studies reported that the virus could not be isolated after day 8 post-symptom onset from respiratory specimens [8,13], but others could isolate the virus from specimens taken up to 18 days after symptom onset [24]. Another study reported that virus culture from specimens with Ct values of more than 34 was unsuccessful [13]. Although these studies gave valuable information on the potential infectivity, the correlation between detectable viral RNA, the virus load, and transmissibility is still unclear and needs further investigation in future studies. This information is absolutely necessary to adapt infection control measures.

Study limitations: The study was a retrospective cohort study with all diagnostic procedures being performed for routine care purposes. For this reason, we reported the analyses depending on the time after symptom onset and included only patients with negative follow-up specimens in the analysis of the duration of virus excretion. There may, however, persist a bias insofar as more severely ill patients may have been sampled more frequently. Secondly, due to the rapidly increasing specimen numbers and shortages in several reagents necessary for RNA extraction and RT-PCR, several commercially available and a laboratory developed RT-PCR methods were used for initial diagnostics. These methods have comparable diagnostic performances, but the Ct values may not be comparable between the different methods. Therefore, follow-up specimens were analyzed with the same assay and the virus load analyses were restricted to specimens analyzed with this assay.

## 5. Conclusions

We reported detailed kinetics of SARS-CoV-2 virus load and virus excretion in URT, LRT, stool, and plasma specimens. LRT specimens are the most appropriate specimen type for the detection of SARS-CoV-2 infection because the frequency and duration of SARS-CoV-2 RNA detection was longest and the virus load was highest. Duration of virus excretion was increased in severe cases of SARS-CoV-2 infection.

## Figures and Tables

**Table 1:** Duration of virus detection since symptom onset.

	Median Duration of Virus Detection (Days Post Symptom Onset)			
	All Patients	ICU	No ICU	*p*-Value
Duration of detection (days)	*n* = 110	*n* = 71	*n* = 39	
Minimum URT	12	13	8	<0.001
Maximum URT	17	19	13	<0.001
	*n* = 45	*n* = 40	*n* = 5	
Minimum LRT	16	17	10	0.02
Maximum LRT	24	25	21	0.09
Duration of detection Ct <33 (days)	*n* = 74	*n* = 42	*n* = 32	
Minimum URT	8.5	11	5	<0.001
Maximum URT	14	16.5	12	0.002
	*n* = 49	*n* = 45	*n* = 4	
Minimum LRT	14	15	9.5	0.10
Maximum LRT	20	20	11	0.006

URT: Upper respiratory tract; LRT: Lower respiratory tract; ICU: Intensive care unit.

## Data Availability

The data presented in this study are available on request from the corresponding author. The data are not publicly available due to privacy restrictions.

## Supplementary Materials

The following are available online at https://www.mdpi.com/2075-4418/11/3/427/s1, Table S1: Patients’ characteristics.

## Abbreviations

CI95	95% confidence interval
ICU	Intensive care unit
LRT	Lower respiratory tract
Max	Maximum
Min	Minimum
RT-PCR	Reverse transcription polymerase chain reaction
SARS-CoV-2	SARS-Coronavirus-2
URT	Upper respiratory tract
