# Supplementary material for: Spatial and Temporal Virus Load Dynamics of SARS-CoV-2: A Single-Center Cohort Study
Source: Diagnostics (Basel). 2021 Mar 3;11(3):427. doi: 10.3390/diagnostics11030427 (PMC7999170; doi:10.3390/diagnostics11030427)
Supplement: Supplementary file 1 [file diagnostics-11-00427-s001.pdf]

Table S1. Patients' characteristics.

Number	Age (Years)	Sex	BMI (kg/m²)	Underlying Disease	Immunosuppression	Intensive Care Unit Admission	Mechanical Ventilation
1	85	M	28	Coronary heart disease, cardiac insufficiency, interstitial pneumopathy, sleep apnoea syndrome	No	Yes	Yes
2	69	M	25	Coronary heart disease, sleep apnoea syndrome	No	Yes	Yes
3	75	M	42	Arterial hypertension	No	Yes	Yes
4	63	F	45	Asthma, sleep apnoea syndrome, type 2 diabetes	No	Yes	Yes
5	60	M	27	Arterial hypertension, coronary heart disease, sleep apnoea syndrome, type 2 diabetes	No	Yes	Yes
6	56	M	37	Arterial hypertension	No	Yes	Yes
7	40	M	33	Arterial hypertension	No	Yes	Yes
8	66	F	26	None	No	Yes	Yes
9	71	M	25	Neurofibroma	No	Yes	Yes
